# Computational Prediction of Inhibitors and Inducers of the Major Isoforms of Cytochrome P450

**DOI:** 10.3390/molecules27185875

**Published:** 2022-09-10

**Authors:** Anastassia Rudik, Alexander Dmitriev, Alexey Lagunin, Dmitry Filimonov, Vladimir Poroikov

**Affiliations:** 1 Department of Bioinformatics, Institute of Biomedical Chemistry, Pogodinskaya Str. 10, Bldg.8, 119121 Moscow, Russia; 2 Department of Bioinformatics, Pirogov Russian National Research Medical University, Ostrovityanova Str. 1, 117513 Moscow, Russia

**Keywords:** CYP, inhibitors, inducers, (Q)SAR models, PASS, GUSAR, drug-like compounds, metabolism, in silico prediction, P450 isoforms, 1A2, 3A4, 2D6, 2C9, 2C19

## Abstract

Human cytochrome P450 enzymes (CYPs) are heme-containing monooxygenases. This superfamily of drug-metabolizing enzymes is responsible for the metabolism of most drugs and other xenobiotics. The inhibition of CYPs may lead to drug–drug interactions and impair the biotransformation of drugs. CYP inducers may decrease the bioavailability and increase the clearance of drugs. Based on the freely available databases ChEMBL and PubChem, we have collected over 70,000 records containing the structures of inhibitors and inducers together with the IC50 values for the inhibitors of the five major human CYPs: 1A2, 3A4, 2D6, 2C9, and 2C19. Based on the collected data, we developed (Q)SAR models for predicting inhibitors and inducers of these CYPs using GUSAR and PASS software. The developed (Q)SAR models could be applied for assessment of the interaction of novel drug-like substances with the major human CYPs. The created (Q)SAR models demonstrated reasonable accuracy of prediction. They have been implemented in the web application P450-Analyzer that is freely available via the Internet.

## 1. Introduction 

Biotransformation of xenobiotics (in other words, drug metabolism) can be described as the biological transformation of external to organism lipophilic nonpolar molecules into more hydrophilic polar metabolites, which in turn are easily excreted from the body. Biotransformation has a significant effect on the pharmacokinetics of most drugs. From the chemical point of view, drug metabolism reactions can be divided into two large categories: oxidative reactions (phase I) and conjugation reactions (phase II) [1]. The human cytochrome P450 enzyme (CYP) family is the main phase I enzymes and contains 57 isoenzymes. CYPs metabolize approximately two thirds of known drugs in humans, with 80% of this process occurring by five isoenzymes—1A2, 2C9, 2C19, 2D6, and 3A4 [2]. Safety is a serious problem for many launched drugs, especially for patients taking multiple medications [1,3]. Adverse drug reactions (ADRs) are among the top 10 leading causes of death, and it is estimated that one hundred thousand deaths per year are attributed to ADRs [1]. ADRs caused by drug–drug interactions (DDIs) can lead to early termination of drug development or even to the withdrawal of drugs from the market; astemizole, cerivastatin, cisapride, terfenadine, and mibefradil are some examples of drugs withdrawn from the market. It is known that most DDIs are mediated through CYP inhibition and induction [3,4]. Such DDIs are manifested by the effect of one drug (the perpetrator drug) on the biotransformation of others (the victim drugs); this may be a slowdown in the case of the inhibition of CYPs or an acceleration in the case of the induction of CYPs; nevertheless, the pharmacological action of coadministered drugs may be altered. In some cases, DDIs can cause more than tenfold increases or decreases in victim drug exposure, with dangerous health effects [5]. Thus, the FDA’s guidance documents prescribe the preliminary screening of drugs for interaction with CYPs [6].

In vitro and in vivo investigations are performed to study the safety and side effects of drugs. Computational (in silico) approaches take much less time to evaluate a large number of compounds both for the known drugs and for new, not yet synthesized drug-like substances. Recently, various computational approaches have been used to create structure–activity relationship (SAR) classification models for predicting drug activity in relation to the interaction with CYPs; a comprehensive description of the methods for DDIs in silico prediction is presented in the review [7]. In silico predictions of interaction with CYPs are made using two approaches: ligand-based and structure-based. Ligand-based predictions of inhibition can be performed by two categories of methods: regression (predicted using IC_50_ or K_i_ values of CYP) and classification (predicted using the category of CYP inhibitory potency). The induction prediction for some CYPs can be performed indirectly by an assessment of the xenobiotic’s interaction with nuclear receptors: the aryl hydrocarbon receptor (AhR), the constitutive androstane receptor (CAR), and the pregnane X receptor (PXR). Structure-based approaches to predicting inhibition of CYPs use CYP structures, docking simulations, and/or molecular dynamics and were not applied for the creation of web services due to the complexity of implementation. Some of the created ligand-based SAR models were implemented as freely available web applications, which predict, based on the structural formula of compounds, their ability to inhibit CYP isoforms important for drug metabolism. They are PreADMET [8], pkCSM [9], SwissADME [10], WhichCyp [11], CYPlebrity [12], vNN-ADMET [13], AdmetSAR [14], and SuperCYPsPred [15]. Despite the good predictive accuracy of these applications, they do not predict CYP-inducing activity and IC_50_ values of the inhibitors. To overcome these limitations, we created the freely available web application P450-Analyzer, which predicts inhibition (including IC_50_ values) and directly predicts the induction of CYPs with reasonable accuracy.

## 2. Results

Using GUSAR [16] and PASS [17] software based on the data from ChEMBL and PubChem database, we created (Q)SAR models for inhibitors and SAR models for inducers of the most important drug-metabolizing isoform of CYPs.

320 QSAR models were built based on the data from ChEMBL by GUSAR using different sets of QNA (Quantitative Neighborhoods of Atoms) and MNA (Multilevel Neighborhoods of Atoms) descriptors and the RBF–SCR (Radial-Basis Function and Self-Consistent Regression) algorithm for each training set with the information about the appropriate isoforms of CYPs. Only those QSAR models in which R^2^ exceeded 0.6 and Q^2^ exceeded 0.5 were selected in the consensus models. The 5-fold cross-validation (5-fold-CV) procedure was used to validate the accuracy of the prediction of QSAR models. The initial datasets were sorted by the ascending mode of pIC_50_ values. After that, the sets were divided into five parts, which were used for the 5-Fold-CV procedure. As a result, different five training and five external test sets for IC_50_ data were created for each isoform. The prediction accuracy of the created consensus QSAR models along with the characteristics of the training sets are represented in Table 1.

Table 1 shows that Q^2^ values in QSAR models for the CYPs 1A2, 2C9, and 3A4 exceed 0.6. The standard deviations between the predicted and experimental data for almost all studied isoforms of CYPs were less than 0.6 and close to 0.5, excluding CYP 1A2 (0.625), which reflects the acceptable accuracy of prediction. RMSE values given on the test sets during 5-fold cross-validation vary from 0.565 (2C19) to 0.682 (1A2). They are less 0.7 and may be considered as applicable. **R^2^** values given on the test sets during 5-fold cross-validation (R^2^_5-Fold-CV_) exceed 0.5 for 1A2, 2C9, and 3A4. R^2^_5-Fold-CV_ for 2C19 and 2D6 are less 0.5, and the prediction results of such models should be treated with caution. Most compounds from the test sets during 5-fold cross-validation fall into the applicability domain (AD). The percent of compounds in AD exceed 95% for all isoforms. 

For all CYP isoforms in the training sets, the pIC_50_ value ranges are characterized by a significant width and include both inactive and very active compounds. The mean values of pIC_50_ are close to 5, which is a threshold used by medicinal chemists to make a division between the active and inactive compounds [18]. Therefore, the created QSAR models may be used in web applications for estimating the degree of activity of potential inhibitors of the CYPs. Taking into account the mean values and the values of standard deviation (Table 1), one may conclude that compounds with prediction results, including pIC_50_ values exceeding 6, may be considered as potential inhibitors of the appropriate CYPs and should by experimentally tested.

Fifteen classification SAR models, depending on the training sets, for prediction of inhibitors for appropriate isoforms of CYPs were built using PASS software. In this case, in addition to a dataset from ChEMBL, we used a dataset from PubChem. The accuracy of prediction for the created SAR models and sources of the training sets are represented in Table 2. The “Total” column represents models, which were built from both ChEMBL and PubChem resources. To calculate the quality of the SAR model, we used the Invariant Accuracy of Prediction (IAP) (which is numerically similar to ROC AUC), calculated by leave-one-out cross-validation procedure (LOO CV) and 20-fold CV. As one may see, for both ChEMBL and PubChem training sets, the best models were obtained for CYP1A2. The combined training set allowed us to improve the quality only for 2D6; in all other cases, the models with PubChem data were better. 

**Figure 1:**
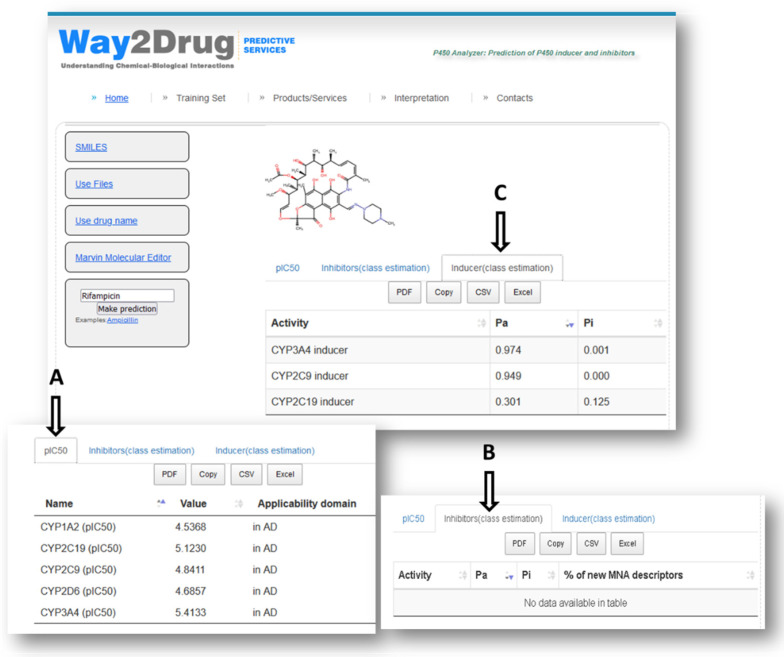
Prediction results for Rifampicin with the created web application.

The created (Q)SAR models were implemented in a new web application (P450-Analyzer), which is freely available at http://www.way2drug.com/p450-analyzer/ (accessed on 16 August 2022). It provides the ability to draw the structures in Marvin Applet, to input SMILES or drug name, or to use the initially prepared MOL file. The prediction results include three tables (Figure 1). The first table is a result of the QSAR models’ application, which represents the numerical estimation of pIC_50_. The highest predicted pIC_50_ values represent the most potent inhibitory activity of compounds for the appropriate CYP isoforms. The second table presents a result of classification models to predict the belonging of inhibitors of CYP isoforms. The table includes the name of the isoenzymes, Pa and Pi values, which are the probabilities “to be inhibitors” and ”to be non-inhibitors“, respectively. For our web application, we used models obtained from both datasets (“Total” column in Table 2). The third table represents the selected activities from the PASSOnline (http://www.way2drug.com/passonline/) (accessed on 16 August 2022); these activities are responsible for the belonging of compounds to CYP inducers. The training set of PASS software is collected from various sources, including commercially available databases, and currently contains over 1.5 million compounds. PASS predicts more than eight thousand activities with an average accuracy about 0.93, estimated in leave-one-out cross-validation. The accuracy of prediction for the selected activities related to CYP inducers is represented in Table 3.

Each table generated in the web application may be downloaded as PDF, CSV, and Excel files.

In order to demonstrate how the web application may be used (Figure 1), we took Rifampicin, which is a well-known inducer of CYPs [19] as a case study. In Figure 1, one can see three tables: table A represents the numerical estimation of pIC50, and tables B and C represent a probabilistic assessment of belonging to inhibitors or inducers of CYPs, respectively. We chose the default threshold Pa>Pi for displaying possible activities. As one can see in Figure 1, in table B, there are no activities, meaning that no inhibitory activity was predicted with Pa>Pi; therefore, Rifampicin could not be classified as a CYP inhibitor. This result coincides with the predicted result of pIC50 values (table A), which are less than 6, and it means that Rifampicin is not a CYP inhibitor. There are three activities in table C. So, Rifampicin is correctly predicted as the inducer of the CYPs 3A4, 2C9, and 2C19. It should be emphasized that Rifampicin was excluded from the training set before the prediction.

## 3. Discussion

CYP-mediated metabolism represents a major route of elimination of many drugs; therefore, information about CYP inhibition and induction is very important to prevent DDIs. We created the web application (P450-Analyzer), which allows estimating the possibility for a drug-like compound to be an inhibitor or inducer of CYPs. Unlike the other existing computational tools, our web application provides an estimation of the IC_50_ values, which allows classifying compounds as strong, moderate, and weak CYP inhibitors. Thus, with the P450-Analyzer, it is possible to perform a more thorough analysis of the potential DDIs. In addition, the P450-Analyzer allows estimating the inducing activity of compounds for five of the most important drug-metabolizing isoforms of CYPs.

## 4. Materials and Methods 

### 4.1. ChEMBL and PubChem

Similar to the CYPlebrity [12], we used the ChEMBL and PubChem databases as a resource for extraction of structures and experimental data for compounds studied on inhibition of CYPs 1A2, 3A4, 2D6, 2C9, and 2C19. The selection criteria were the same as in the publication [12]. 

From PubChem, we took bioassay 1851 dataset [20], which was downloaded as CSV table; then, SMILES notations for the selected compounds were retrieved by querying PubChem’s download services. Compounds with “Pubchem_activity_outcome” = “active” supported by a “Complete curve” were assigned to the “inhibitors” class. Compounds with “Pubchem_activity_outcome” = “inactive” were marked as “non-inhibitors”. 

On the basis of ChEMBL, we created two types of training sets. The first type was used to build classification models and contains qualitative information; the compounds were classified as inhibitors or noninhibitors, based on IC_50_ values and percentage of inhibition. The entries with IC_50_ lower than 10,000 nM were defined as inhibitors if the field ”standard relation“ containing any of the signs “=”, “≤”, or “<”. The entries with IC_50_ exceeding 20,000 nM were defined as noninhibitors if the field ”standard relation“ containing one of signs “=”, “≥”, or “>”. In addition, the entries with percentage of inhibition > 50% were defined as inhibitors and otherwise as noninhibitors. The conflicting data (if the same structure was classified as inhibitor and noninhibitor) were removed from the training set.

The second type of training sets was used to build QSAR models and contained quantitative data on enzyme inhibition. For these types of training sets, only IC_50_ values with the field “standard relation” containing sign “=” were considered. The data in the training sets for double records of the same structures were recalculated as median values. IC50 values in nM were converted to pIC_50_ = −log_10_^(M)^.

After the data selection and filtration of the training sets containing structures and appropriate information (pIC_50_ or belonging to inhibitors), the SDfiles were created for the appropriate CYP isoforms. Then, these training sets were used in PASS and GUSAR software to build the appropriate (Q)SAR models. Characteristics of the training sets for development of classification models are represented in Table 4.

**Figure 2:**
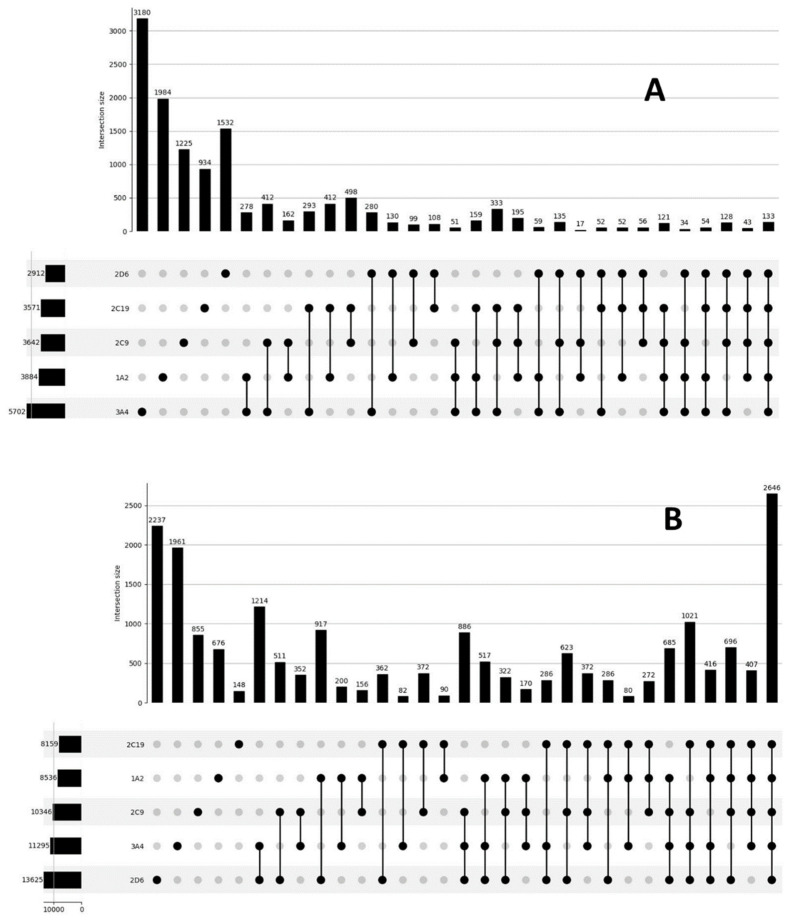
UpSet Plot showing how inhibitors (**A**) and noninhibitors (**B**) of different isoenzymes intersect.

The intersections of the training sets in “Total” model are shown in Figure 2. As can be seen, the compounds presented in the sets usually inhibit only one isoenzyme; 133 compounds inhibit all isoenzymes. Among the noninhibitors, most compounds do not inhibit any of the enzymes.

**Figure 3:**
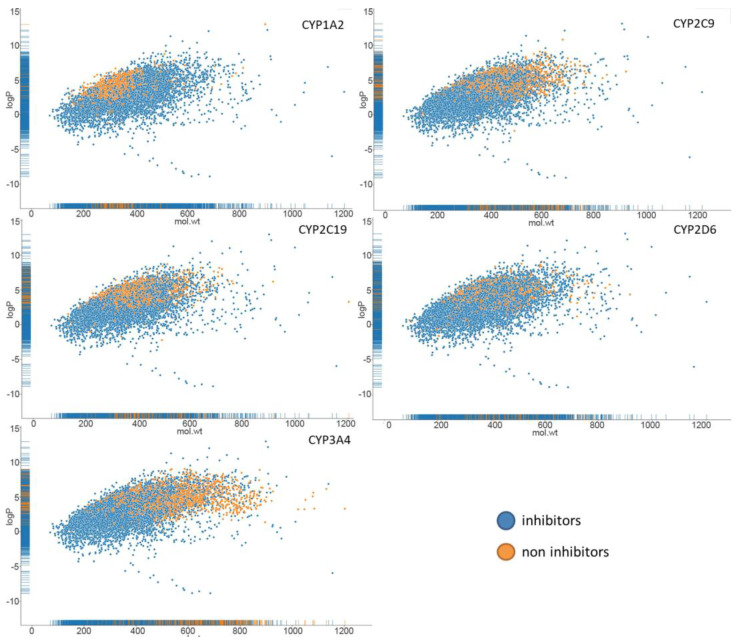
The distribution of the calculated logP vs. molecular weight for inhibitors and noninhibitors of isoenzymes.

Additionally, “Total” model chemical space of inhibitors and noninhibitors was shown by molecular weight as X-axis and logP, calculated by RdKit, as Y-axis (see Figure 3). 

### 4.2. GUSAR

GUSAR software [16,21] was used to create the QSAR models predicting the inhibition of CYP isoforms on the basis of the structural formula of compounds. GUSAR is based on the representation of the compound structure by QNA and MNA descriptors [16,17] A combined RBF–SCR algorithm was used to build the relationships between the descriptors and biological activity [22]. To improve the accuracy of prediction, GUSAR allows to create a consensus model; the final predicted value for each activity/end point is estimated by including the weighted average of the predicted values from the set of QSAR models [23]. The value obtained from each model is weighted by the similarity value calculated in the estimation of its applicability domain. For applicability domain calculation in GUSAR, three different approaches are used for each model: similarity, leverage, and accuracy assessment [23].

### 4.3. PASS 

PASS software [17] predicts the profile of biological activity based on advanced naïve Bayes classifier. The molecular structures of compounds are described by Multilevel Neighborhoods of Atom (MNA) descriptors. Accuracy of prediction is estimated in PASS by leave one-out cross-validation procedure using the IAP criterion. IAP is a probability that is randomly selected from an independent test set; positive and negative examples will be correctly classified [14]. The PASS output is a ranked list of activities with estimates the probabilities ”to be active“ Pa and ”to be inactive“ Pi. The activities with Pa > Pi are considered as possible. The percent of new MNA descriptors for the tested molecule is used in the web application for assessment of the applicability domain; the higher the percent of new MNA descriptors, the less the molecule structure is appropriate for the model. The tested molecules with up to 25% of new MNA descriptors are in the applicability domain [24].

## Figures and Tables

**Table 1:** Characteristics of the training sets and prediction accuracy of consensus QSAR models for CYP inhibitors.

CYP	N_cmp_	Interval of Values, pIC_50_	Mean Value, pIC_50_	N_mdls_	Training Sets			5-Fold-CV		
					R^2^	Q^2^	SD	R^2^	RMSE	AD, %
1A2	1216	[1.6:8.8]	5.47	65	0.999	0.680	0.625	0.614	0.682	99.4
2C9	1657	[1.5:9.0]	5.28	72	0.994	0.609	0.512	0.508	0.565	95.6
2C19	930	[2.8:8.3]	5.16	68	0.992	0.501	0.519	0.348	0.588	99.0
2D6	1588	[1.2:9.2]	5.32	59	0.972	0.566	0.567	0.480	0.619	97.1
3A4	3299	[1.4:10.3]	5.39	89	0.992	0.640	0.528	0.589	0.562	98.2

**N_cmp_**—number of compounds in the training set; **N_mdls_**—number of QSAR models in the appropriate consensus model; **SD**—standard deviation; **5-Fold-CV**—results given on the test sets during 5-fold cross-validation; **AD**, **%**—% of compounds from the test sets in applicability domain.

**Table 2:** Prediction accuracy (IAP) for classification models.

CYP	ChEMBL		PubChem		Total	
	LOO CV	20-Fold CV	LOO CV	20-Fold CV	LOO CV	20-Fold CV
1A2	0.884	0.884	0.937	0.937	0.923	0.922
2D6	0.873	0.873	0.861	0.861	0.891	0.891
2C9	0.827	0.826	0.875	0.875	0.855	0.854
2C19	0.816	0.813	0.879	0.878	0.856	0.856
3A4	0.845	0.845	0.896	0.895	0.871	0.870

**Table 3:** Characteristics of selected activities from PASSOnline.

Activities	N_pos_	IAP, LOO CV	IAP, 20-Fold CV
1A2 inducer	26	0.907	0.905
2C9 inducer	28	0.846	0.846
2C19 inducer	8	0.840	0.839
2D6 * inducer	4	0.604	-
3A4 inducer	78	0.893	0.879

**N_pos_**—number of inducers in the training set; **IAP, LOO CV**—Invariant Accuracy of Prediction calculated by leave one-out cross-validation procedure. * The number of the four finding CYP 2D6 inducers is too small to create good SAR model.

**Table 4:** Characteristics of the training sets for development of classification models.

CYP	ChEMBL		PubChem		Total	
	N_pos_	N_neg_	N_pos_	N_neg_	N_pos_	N_neg_
1A2	1098	2183	2035	3680	3141	8536
2D6	1955	3414	999	10,249	2912	13,625
2C9	2074	2750	1586	7635	3642	10,346
2C19	1050	1706	2538	6484	3571	8159
3A4	3836	4501	1890	6829	5702	11,295

**N_pos_**—number of inducers in the training set; **IAP, LOO CV**—Invariant Accuracy of Prediction, calculated by leave one-out cross-validation procedure

## Data Availability

The data presented in this study are available in Supplementary Materials at: https://www.mdpi.com/article/10.3390/molecules27185875/s1.

## Supplementary Materials

The following supporting information can be downloaded as archive containing SDfiles at: https://www.mdpi.com/article/10.3390/molecules27185875/s1.

## Sample Availability

Samples of the compounds are not available from the authors.
